# Increased Ratio of Global *O*-GlcNAcylation to Tau Phosphorylation at Thr212 Site Is Associated With Better Memory Function in Patients With Type 2 Diabetes

**DOI:** 10.3389/fphys.2019.00110

**Published:** 2019-02-14

**Authors:** Rong Huang, Sai Tian, Jing Han, Rongrong Cai, Hongyan Lin, Dan Guo, Jiaqi Wang, Shaohua Wang

**Affiliations:** ^1^Department of Endocrinology, Affiliated Zhongda Hospital, Southeast University, Nanjing, China; ^2^School of Medicine, Southeast University, Nanjing, China

**Keywords:** tau protein, *O*-GlcNAcylation, phosphorylation, mild cognitive impairment, type 2 diabetes mellitus

## Abstract

**Objective:** Aberrant *O*-GlcNAc modification has been implicated in type 2 diabetes mellitus (T2DM) and the pathogenesis of neurodegenerative diseases via competition with tau phosphorylation. We aimed to investigate the association between global *O*-GlcNAcylation, tau phosphorylation levels and mild cognitive impairment (MCI) in the whole blood of patients with T2DM.

**Methods:** Sociodemographic, clinical characteristics and cognitive performances of the enrolled T2DM subjects were extensively assessed. Global *O*-GlcNAcylation and tau phosphorylation levels in the whole blood were also determined using Western blot.

**Results:** Forty-eight T2DM subjects, including 24 with MCI and 24 with normal cognition, were enrolled in this study. Compared with cognitively normal controls, T2DM with MCI subjects displayed decreased global *O*-GlcNAcylation level, but increased tau phosphorylation levels (all *p* < 0.05). To reflect the combined effect, the ratios of global *O*-GlcNAcylation to tau phosphorylation levels, including specific sites, such as Ser396, Ser404, Thr212, and Thr231, were all significantly decreased in MCI subjects (all *p* < 0.05). Further multivariable logistic regression analysis revealed that high glycated hemoglobin A1c was an independent risk factor, whereas increased *O*-GlcNAc/p-T212 was an independent protective factor for MCI in patients with T2DM (odds ratio [OR] = 2.452, 95% confidence interval [CI] 1.061–5.668, *p* = 0.036; OR = 0.028, 95%CI 0.002–0.388, *p* = 0.008, respectively). With regard to each cognitive domain, *O*-GlcNAc/p-T212 was positively correlated with the score of Auditory Verbal Learning Test-delayed recall (*r* = 0.377, *p* = 0.010).

**Conclusion:** Our study suggests that increased ratio of global *O*-GlcNAcylation to tau phosphorylation at Thr212 site in the whole blood is associated with decreased risk of MCI, especially with better memory function in T2DM subjects.

**Clinical Trial Registration:** www.ClinicalTrials.gov, identifier ChiCTR-OCC-15006060.

## Introduction

Current estimates indicated that 415 million adults are diagnosed with diabetes worldwide in 2015 (Ogurtsova et al., 2017). Among these adults, 90% manifested type 2 diabetes mellitus (T2DM). T2DM is a multifactorial metabolic disorder that can cause several acute and chronic complications, mainly including diabetic ketoacidosis, diabetic hyperosmotic coma, cardiovascular and cerebrovascular diseases, and microvascular diseases (Zheng et al., 2018). Previous studies suggested that T2DM is an independent risk factor for mild cognitive impairment (MCI) and dementia (Cheng et al., 2012; Gao et al., 2016), and also progression from MCI to dementia (Li et al., 2016; Ciudin et al., 2017). Moreover, the annual conversion rate of MCI to Alzheimer’s disease (AD) is 10–15%, and 80% of MCI patients are converted to AD within 6 years, which will cause huge family and social burdens (Tiermey et al., 1996). Therefore, it is urgent to provide early prevention and intervention of MCI in patients of T2DM. The underlying mechanisms of T2DM-related cognitive dysfunction are complex and include chronic hyperglycemia (Kim D. J. et al., 2016), recurrent hypoglycemia (Sheen and Sheu, 2016; Mehta et al., 2017), insulin resistance (IR) (Ma et al., 2015), β-amyloid aggregation, and tau protein hyperphosphorylation (Baglietto-Vargas et al., 2016; Mittal and Katare, 2016). However, the exact mechanisms require further studies.

*O*-*N*-acetylglucosaminylation (*O*-GlcNAcylation) is a ubiqui-tous and dynamic posttranslational modification regulated by only two known enzymes, namely, *O*-linked β-*N*-acetyl-glucosamine transferase (OGT) and *O*-linked β-*N*-acetylglucosaminidase (*O*-GlcNAcase, OGA) (Yang and Qian, 2017). OGT transfers *O*-linked *N*-acetylglucosamine (*O*-GlcNAc) to the hydroxyl group of serine (Ser) or threonine (Thr) residues of certain cytoplasmic, nuclear and mitochondrial proteins, whereas OGA removes it from proteins. To date, thousands of *O*-GlcNAc-modified proteins reportedly regulate many cellular pathways, such as epigenetics, gene expression, translation, protein degradation, signal transduction, mitochondrial bioenergetics, cell cycle, and protein localization (Zachara et al., 2015). *O*-GlcNAc pathway utilizes uridine diphosphate *N*-acetylglucosamine (UDP-GlcNAc) from the hexosamine biosynthetic pathway. Thus, previous studies linked it to circulating glucose levels. For example, aberrant protein *O*-GlcNAc modification has been associated with hyperglycemia and IR (Park et al., 2010; Myslicki et al., 2014). Moreover, increased expression of *O*-GlcNAcylation in erythrocyte or leukocyte proteins (particularly granulocyte) is considered as a unique marker for early and efficient detection of T2DM (Wang et al., 2009; Springhorn et al., 2012). In addition, *O*-GlcNAcylation has been also implicated in the pathogenesis of diabetic complications, such as but not limited to retinopathy, nephropathy, and vascular disease (Peterson and Hart, 2016).

*O*-GlcNAcylation and phosphorylation modify Ser and/or Thr side chains of substrate proteins. Consequently, *O*-GlcNAcylation can compete with phosphorylation at certain sites of various proteins, including microtubule-associated protein tau (Hart et al., 2011). Studies demonstrated that the formation of neurofibrillary tangles, in which the major components are abnormally hyperphosphorylation tau proteins, is one of the defining pathological features of AD (Grundke-Iqbal et al., 1986). As a consequence, aberrant tau *O*-GlcNAcylation has been implicated in the pathogenesis of Alzheimer and neurodegenerative diseases. In human postmortem brain tissues, tau protein was demonstrated to be modified by *O*-GlcNAcylation, and protein *O*-GlcNAcylation level in AD brain was lower than that in healthy controls (Liu F. et al., 2004). Evidence from an animal study indicated an imbalance between tau phosphorylation and *O*-GlcNAcylation in the hippocampus of 3xTg-AD mice (Gatta et al., 2016). Intervention studies also suggested that increased *O*-GlcNAcylation may prevent (β-amyloid plaque formation and pathological tau aggregation, thereby rescuing cognitive impairment in transgenic mouse models (Kim et al., 2013; Graham et al., 2014; Yuzwa et al., 2014).

However, knowledge is lacking on the relationships among O-GlcNAcylation, tau phosphorylation level and cognitive functions in peripheral blood samples from patients with T2DM. Given the researches that suggested a link of O-GlcNAcylation with phosphorylation modification and cognition, as well as IR and diabetes, we hypothesized that the imbalance between O-GlcNAcylation and tau phosphorylation may be involved in diabetes-associated cognitive decline. Herein, we aimed to investigate the association among global O-GlcNAcylation, tau phosphorylation levels, MCI, and the different cognitive domain performances in the whole blood of patients with T2DM.

## Materials and Methods

### Ethics Statement

This study was conducted in the Department of Endocrinology, Affiliated Zhongda Hospital of Southeast University. The study protocol and informed consent documents were approved by the Research Ethics Committee of the Affiliated Zhongda Hospital of Southeast University. All enrolled subjects were informed about the study and gave written consent.

### Subject Recruitment

Subjects were recruited from the Department of Endocrinology, Affiliated Zhongda Hospital of Southeast University from January 2017 to October 2017. All patients with T2DM were diagnosed according to the World Health Organization (1999) criteria (Alberti and Zimmet, 1998). They were aged between 45 and 75 years with a history of diabetes > 3 years. Patients with any of the following criteria were excluded: (1) presence of acute diabetic complications, including diabetic ketoacidosis, hyperosmolar non-ketotic diabetic coma, diabetic lacatocidosis, and severe hypoglycemia; (2) presence of acute cardiovascular or cerebrovascular accident, such as acute myocardial infarction, and acute cerebral hemorrhage; (3) any known history of central nervous system diseases that could affect cognition, including stroke (Hachinski ischemic score [HIS] ≥ 4), head trauma, epilepsy, major anxiety and depression; (4) history of alcoholism or drug abuse within 2 months; (5) the presence of thyroid dysfunction or other severe medical illness, such as cancer, anemia and serious infection; and (6) history of severe visual or hearing loss. MCI was diagnosed based on criteria established by the National Institute on Aging-Alzheimer’s Association workgroups (Albert et al., 2011). The criteria included the following: (1) a reported decline in cognitive function (self/informant/clinician report); (2) objective evidence of impairment in one or more cognitive domains, which were assessed by the Montreal Cognitive Assessment (MoCA) in this study; (3) preservation of normal activities of daily living (ADL), measured by ADL questionnaire; and (4) the absence of dementia (based on the Diagnostic and Statistical Manual of Mental Disorders-IV criteria).

### Sociodemographic and Clinical Characteristic Collection

Standard sociodemographic and clinical characteristics of the enrolled subjects were collected at study entry, including age, gender, educational level, weight, height, blood pressure (BP), and diabetes duration. Body mass index (BMI) was calculated as body weight (kg) divided by the square of body height (m^2^). Hypertension was defined as BP ≥ 140/90 mmHg and/or use of antihypertensive medication. Lifestyle risk factors such as smoking and alcohol history were also included. The laboratory data comprised fasting blood glucose (FBG), fasting C-peptide (FCP), glycated hemoglobin A1c (HbA1c), triglycerides, total cholesterol, high density lipoprotein cholesterol, low density lipoprotein cholesterol, serum creatinine (SCr), blood urea nitrogen (BUN), and uric acid (UA). IR was evaluated according to the modified homeostatic model assessment of insulin resistance (HOMA-IR) formula based on FCP: FBG (mmol/L) × FCP (ng/mL)/22.5 (Kim J. D. et al., 2016). Data on antidiabetic drug usage, including metformin and insulin, were also collected in this study.

### Cognitive Performance Assessment

Cognitive performances were assessed through a battery of neuropsychological scales designed to evaluate the performance in approximately 60 min across an array of cognitive domains including memory, executive function, visual spatial processing, attention, and information processing speed. The MoCA test was used to assess overall cognitive function ranging from 0 to 30 score and also regarded as a highly sensitive screening tool of MCI in patients with T2DM (Alagiakrishnan et al., 2013). The normal MoCA score was ≥26, with one point added if the subject had less than 12 years of formal education (Nasreddine et al., 2005). Other tests included Digit Span Test, Verbal Fluency Test, Clock Drawing Test (CDT), Trail Making Test-A and B, and Auditory Verbal Learning Test (AVLT). HIS, clinical dementia rating (CDR), and self-rating depression scale were also obtained. All neuropsychological tests were performed by a skilled neuropsychiatrist from the Department of Neurology, Affiliated Zhongda Hospital of Southeast University, and all the subjects were blinded to the study design.

### Western Blot

Whole blood samples were collected from the enrolled subjects by venipuncture in anticoagulant-free tubes, and then immediately stored at -80°C. In preparation for Western blot, blood samples were homogenized in a lysis buffer, which consists of 50 mM Tris (pH 7.4), 150 mM NaCl, 1% Triton X-100, 1% sodium deoxycholate, 0.1% sodium dodecyl sulfate (SDS), and various protease and phosphatase inhibitors, including phenylmethanesulfonyl fluoride, sodium pyrophosphate, and β-glycerophosphate. The samples were then centrifuged at 12,000 *g* at 4°C for 10 min, and the lysate was recovered and diluted 20 times. Protein concentrations were determined by the bicinchoninic acid assay according to the manufacturer’s protocol (NanJing KeyGen Biotech Co., Ltd.). 20 μg total protein was resolved on SDS-polyacrylamide gel electrophoresis gels and blotted to polyvinylidene fluoride (PVDF) membranes. PVDF membranes were then blocked by 5% non-fat milk in Tris-buffered saline with 0.1% Tween 20 (TBST) at room temperature for 1 h, and incubated overnight at 4°C with appropriate primary antibodies. Primary antibodies included anti-*O*-linked *N*-acetylglucosamine [RL2] (1:1000; Abcam, ab2739), anti-OGT/*O*-linked *N*-acetylglucosamine transferase (1:5000; Abcam, ab177941), anti-MGEA5 (1:5000; Abcam, ab124807), anti-Tau [Tau-5] (1:800; Abcam, ab80579), anti-Tau (phosphor S396) (1:10000; Abcam, ab109390), anti-Tau (phosphor S404) (1:1000; Abcam, ab92676), anti-Tau (phosphor T212) (1:1000; Abcam, ab4842), and anti-Tau (phosphor T231) (1:5000; Abcam, ab151559) with glyceraldehyde 3-phosphate dehydrogenase (anti-GAPDH) (1:3000; CMCTAG, AT0002) serving as the control. Secondary antibodies were as follows: goat anti-mouse IgG horseradish peroxidase (HRP) (1:50000; MyBioScience, MKA001A) and goat anti-rabbit IgG HRP (1:25000; MyBioScience, MKA003A). Signals were detected with chemiluminescence (Millipore, WBKLS0500) and then exposed using MiniChemi^TM^610 Imaging and Analysis System (Beijing Sage Creation Science Co., Ltd.).

### Statistical Analyses

Densitometry data of global *O*-GlcNAc, OGT, OGA, and tau phosphorylation levels were analyzed by the Image J program. Data are presented as mean ± standard deviation/standard error of mean (SEM), median (interquartile range) or *n* (percentage). Student’s *t*-test, non-parametric Mann–Whitney *U* test or Chi-square test was used to determine differences of clinical parameters, global *O*-GlcNAcylation level, its key enzyme levels, and tau phosphorylation levels, as well as ratios of global *O*-GlcNAcylation to tau phosphorylation levels between T2DM with MCI patients and those with normal cognition subjects. A simple logistic regression model was first used to explore so-called independent risk or protective factors for MCI in patients with MCI. Then, forward stepwise multivariable regression analysis was used to explore the “strongest” factors affecting the MCI presence. Spearman correlation analyses were performed to explore the relationships of the ratio of global *O*-GlcNAcylation to tau phosphorylation levels with other sociodemographic and clinical characteristics. Furthermore, we conducted partial correlation analyses to assess the relationships between the ratio of global *O*-GlcNAcylation to tau phosphorylation levels and different cognitive domain performances. All statistical analyses were performed using SPSS version 22.0 (SPSS Inc., Chicago, IL, United States), and *p*-value < 0.05 (two-tailed) was considered statistically significant.

## Results

### Sociodemographic, Clinical Characteristics and Cognitive Performances

This study enrolled a total of 48 T2DM subjects, including 24 persons with MCI and 24 cognitively normal controls. The detailed sociodemographic, clinical characteristics and cognitive performances of the participants are summarized in Table 1. The MCI and control group were well matched in age, gender and education levels, as well as the prevalence of smoking, alcohol, hypertension and metformin and insulin usage (all *p* > 0.050). Compared with cognitively normal controls, T2DM with MCI subjects displayed elevated HbA1c and FBG, while decreased FCP and UA (all *p* < 0.05). No significant differences were observed including BMI, diabetes duration, HOMA-IR (FCP), blood lipids, SCr and BUN (all *p* > 0.05). Moreover, T2DM with MCI patients showed significantly poorer overall and different domains of cognitive performances than control subjects (all *p* < 0.05, except the *p*-value for CDT).

**Table 1:** Sociodemographic, clinical characteristics and cognitive performances.

	MCI group	Control group
Characteristic	(*n* = 24)	(*n* = 24)	*p*-value
Age (years)	60.67 ± 6.92	61.25 ± 6.77	0.769^a^
Female, *n* (%)	14 (58.33)	9 (37.50)	0.149^c^
Education Levels (years)	9.50 (9.00–12.00)	11.50 (8.25–12.00)	0.800^b^
Smoking, *n* (%)	11 (45.83)	9 (37.50)	0.558^c^
Alcohol, *n* (%)	8 (33.33)	5 (20.83)	0.330^c^
BMI (kg/m^2^)	25.19 ± 3.45	26.01 ± 3.36	0.409^a^
Hypertension, *n* (%)	17 (70.83)	12 (50.00)	0.140^c^
Diabetes duration (years)	10.00 (8.25–15.75)	10.00 (7.00–13.00)	0.367^b^
Metformin usage, *n* (%)	21 (87.50)	16 (66.67)	0.086^c^
Insulin usage, *n* (%)	17 (70.83)	13 (54.17)	0.233^c^
HbA1c (%)	9.25 ± 1.27	7.95 ± 0.90	<0.001^a^
FBG (mmol/L)	9.68 ± 2.11	7.83 ± 1.62	0.001^a^
FCP (ng/mL)	0.46 (0.33–0.55)	0.58 (0.43–0.83)	0.021^b^
HOMA-IR (FCP)	0.20 (0.13–0.27)	0.21 (0.15–0.28)	0.353^b^
TG (mmol/L)	1.62 (0.89–2.33)	1.34 (0.97–1.98)	0.688^b^
TC (mmol/L)	4.87 ± 1.03	4.38 ± 1.24	0.149^a^
HDL (mmol/L)	1.22 ± 0.27	1.09 ± 0.27	0.102^a^
LDL (mmol/L)	3.04 ± 0.93	2.70 ± 0.88	0.206^a^
SCr (μmol/L)	68.96 ± 19.60	70.28 ± 20.09	0.819^a^
BUN (mmol/L)	5.70 ± 1.15	5.95 ± 1.26	0.469^a^
UA (μmol/L)	282.33 ± 65.91	336.92 ± 98.24	0.029^a^
Neuropsychological tests			
MoCA	24.00 (22.00–25.00)	28.00 (27.00–29.00)	<0.001^b^
DST	11.00 (10.00–11.75)	12.00 (11.00–13.00)	0.023^b^
VFT	14.50 (13.00–18.00)	18.00 (15.25–20.00)	0.012^b^
CDT	4.00 (3.00–4.00)	4.00 (3.00–4.00)	0.596^b^
TMT-A	68.50 (56.50–87.50)	55.00 (45.50–71.00)	0.020^b^
TMT-B	171.50 (116.50–215.00)	123.00 (90.25–159.75)	0.042^b^
AVLT-immediate recall	16.00 (14.00–18.75)	19.00 (15.25–23.50)	0.041^b^
AVLT-delayed recall	5.00 (3.00–6.00)	6.00 (5.00–8.75)	0.016^b^


*Data are presented as n (%), mean ± standard deviation, or median (interquartile range) as appropriate.*

*^*a*^Student’s t-test for comparison of normally distributed quantitative variables between MCI group and control group.*

*^b^Mann–Whitney U test for comparison of asymmetrically distributed quantitative variables between MCI group and control group.*

*^*c*^χ^*2*^ test for comparison of qualitative variables between MCI group and control group.*

*MCI, mild cognitive impairment; BMI, body mass index; HbA1c, glycosylated hemoglobin; FBG, fasting blood glucose; FCP, fasting C-peptide; HOMA-IR(FCP): replacing fasting insulin with FCP in the homeostasis model assessment of insulin resistance formula; TG, triglyceride; TC, total cholesterol; HDL, high-density lipoprotein; LDL, low-density lipoprotein; SCr, serum creatinine; BUN, blood urea nitrogen; UA, uric acid; MoCA, Montreal Cognitive Assessment; DST, Digit Span Test; VFT, Verbal Fluency Test; CDT, Clock Drawing Test; TMT, Trail Making Test; AVLT, Auditory Verbal Learning Test*.

### Global *O*-GlcNAcylation and Tau Phosphorylation Levels Between the MCI and Control Subjects

**FIGURE 1:**
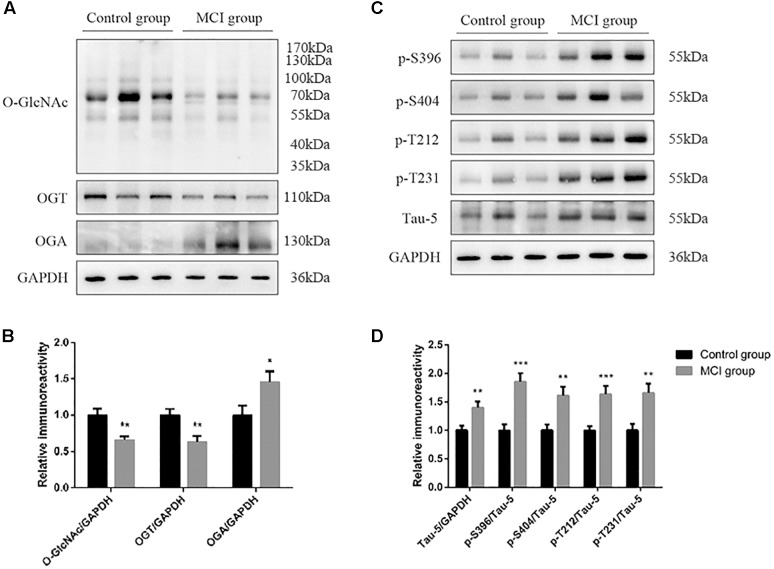
Global *O*-GlcNAcylation and tau phosphorylation levels in the whole blood of T2DM subjects. **(A)** Representative Western blot analysis showing that global *O*-GlcNAcylation and OGT expression are decreased, while OGA expression are increased in T2DM with MCI compared to those with normal cognition. **(B)** Quantitation of Western blot analysis **(A)**. **(C)** Representative Western blot analysis showing that total tau level, as well as phosphorylation of tau protein at specific sites including Ser396, Ser404, Thr212, and Thr231 are increased in T2DM with MCI compared to those with normal cognition. **(D)** Quantitation of Western blot analysis **(C)**. All data represents *n* = 48, and are means ± standard error of mean. ^∗^*p* < 0.05, ^∗∗^*p* < 0.01, ^∗∗∗^*p* < 0.001.

**FIGURE 2:**
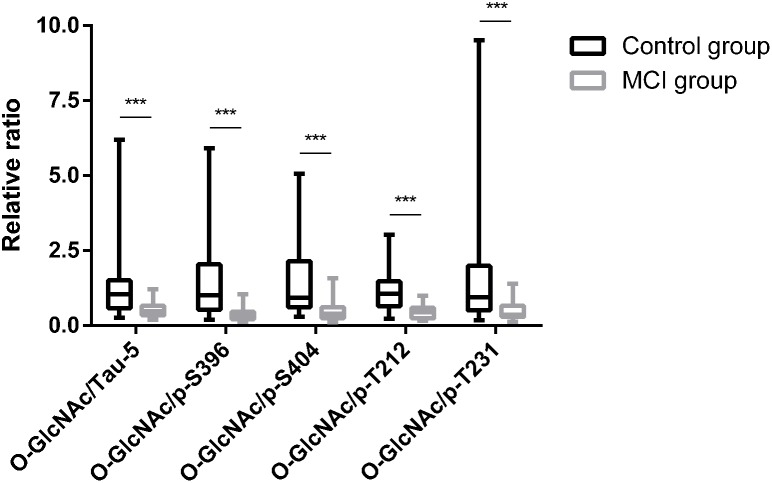
Relative ratio of global *O*-GlcNAcylation to tau phosphorylation levels in T2DM subjects. *O*-GlcNAc/Tau-5, *O*-GlcNAc/p-S396, *O*-GlcNAc/p-S404, *O*-GlcNAc/p-T212, and *O*-GlcNAc/p-T231 were all decreased in T2DM with MCI subjects in comparison to control subjects. All data represents *n* = 48, and are median (interquartile range). ^∗∗∗^*p* < 0.001.

Global *O*-GlcNAcylation and tau phosphorylation levels were determined by western blot and analyzed by densitometry (Figure 1). Compared to T2DM with cognitively normal controls, global *O*-GlcNAcylation level was lower in MCI subjects (*p* = 0.012) (Figure 1A,B). Moreover, there was a significant decrease in OGT expression, but an increase in OGA expression in the MCI group (both *p* < 0.05) (Figure 1A,B). Results also showed that the decrease in global *O*-GlcNAcylation level was accompanied by an increase in total tau level, as well as hyperphosphorylation of tau protein at specific sites including Ser396, Ser404, Thr212 and Thr231 (all *p* < 0.05) (Figure 1C,D). In order to reflect the combined effect and to magnify the effect, we performed relative ratios of global *O*-GlcNAcylation to tau phosphorylation levels, and found that *O*-GlcNAc/Tau-5, *O*-GlcNAc/p-S396, *O*-GlcNAc/p-S404, *O*-GlcNAc/p-T212, and *O*-GlcNAc/p-T231 were all decreased in T2DM with MCI subjects in comparison to control subjects (all *p* < 0.05) (Figure 2).

### Exploration of Risk Factors for MCI in T2DM Patients

To explore risk factors for MCI in T2DM patients, we first conducted a simple logistic regression analysis via entering all sociodemographic and clinical characteristics. The results showed that T2DM subjects with higher HbA1c and FBG were associated with increased risk of MCI, while increased FCP, UA, *O*-GlcNAc/Tau-5, *O*-GlcNAc/p-S396, *O*-GlcNAc/p-S404, *O*-GlcNAc/p-T212, and *O*-GlcNAc/p-T231 were associated with decreased risk of MCI (all *p* < 0.05) (Table 2). Further forward stepwise multivariable logistic regression analysis revealed that high HbA1c was an independent risk factor for MCI, while increased *O*-GlcNAc/p-T212 was an independent protective factor for MCI in T2DM patients (OR = 2.452, 95%CI 1.061–5.668, *p* = 0.036; OR = 0.028, 95%CI 0.002–0.388, *p* = 0.008, respectively).

**Table 2:** Exploration of risk factors for MCI in T2DM patients.

	β	SE of β	*p*-value	OR	95%CI
HbA1c (%)	1.106	0.358	0.002	3.023	1.500–6.092
FBG (mmol/L)	0.568	0.205	0.006	1.765	1.181–2.637
FCP (ng/mL)	-2.714	1.280	0.034	0.066	0.005–0.814
UA (umol/L)	-0.010	0.005	0.042	0.990	0.981–1.000
*O*-GlcNAc/Tau-5	-3.325	1.100	0.003	0.036	0.004–0.311
*O*-GlcNAc/p-S396	-3.752	1.260	0.003	0.023	0.002–0.277
*O*-GlcNAc/p-S404	-2.173	0.877	0.013	0.114	0.020–0.635
*O*-GlcNAc/p-T212	-4.209	1.301	0.001	0.015	0.001–0.190
*O*-GlcNAc/p-T231	-2.103	0.813	0.010	0.122	0.025–0.601


*MCI, mild cognitive impairment; β, regression coefficient; SE, standard error; OR, odds ratio; CI, confidence interval for odds ratio; HbA1c, glycosylated hemoglobin; FBG, fasting blood glucose; FCP, fasting C-peptide; UA, uric acid*.

### Relationships of *O*-GlcNAc/p-T212 With Different Cognitive Domain Performances in T2DM Patients

The Spearman correlation analyses revealed that *O*-GlcNAc/p-T212 was negatively associated with HbA1c and FBG (*r* = -0.346, *p* = 0.016; *r* = -0.329, *p* = 0.023, respectively). No significant associations were found between *O*-GlcNAc/p-T212 and other sociodemographic and clinical characteristics (all *p* > 0.05). Further partial correlation analyses showed that *O*-GlcNAc/p-T212 was positively associated with MoCA after adjustment for HbA1c and FBG (*r* = 0.397, *p* = 0.006). With regard to each cognitive domain, *O*-GlcNAc/p-T212 was positively correlated with the score of AVLT-delayed recall, which represent delayed verbal learning and memory functions (*r* = 0.377, *p* = 0.010) (Table 3).

**Table 3:** Relationships of *O*-GlcNAc/p-T212 with other clinical characteristics and different cognitive domains performances in T2DM patients.

	*O*-GlcNAc/p-T212	
	*r*	*p*-value
Age (years)	0.077	0.604^a^
Education levels (years)	-0.019	0.897^a^
BMI (kg/m^2^)	0.239	0.102^a^
Diabetes duration (years)	-0.065	0.659^a^
HbA1c (%)	-0.346	0.016^a^
FBG (mmol/L)	-0.329	0.023^a^
FCP (ng/mL)	0.127	0.389^a^
HOMA-IR (FCP)	-0.050	0.734^a^
TG (mmol/L)	0.112	0.450^a^
TC (mmol/L)	-0.143	0.334^a^
HDL (mmol/L)	-0.245	0.094^a^
LDL (mmol/L)	-0.194	0.187^a^
SCr (μmol/L)	0.265	0.069^a^
BUN (mmol/L)	-0.062	0.674^a^
UA (μmol/L)	0.185	0.207^a^
MoCA	0.397	0.006^b^
DST	0.038	0.803^b^
VFT	0.197	0.189^b^
CDT	0.104	0.491^b^
TMT-A	0.053	0.725^b^
TMT-B	0.053	0.729^b^
AVLT-immediate recall	0.215	0.152^b^
AVLT-delayed recall	0.377	0.010^b^


*^a^Spearman correlation.*

*^*b*^Partial correlation after adjustment for HbA1c and FBG.*

*MCI, mild cognitive impairment; BMI, body mass index; HbA1c, glycosylated hemoglobin; FBG, fasting blood glucose; FCP, fasting C-peptide; HOMA-IR(FCP): replacing fasting insulin with FCP in the homeostasis model assessment of insulin resistance formula; TG, triglyceride; TC, total cholesterol; HDL, high-density lipoprotein; LDL, low-density lipoprotein; SCr, serum creatinine; BUN, blood urea nitrogen; UA, uric acid; MoCA, Montreal Cognitive Assessment; DST, Digit Span Test; VFT, Verbal Fluency Test; CDT, Clock Drawing Test; TMT, Trail Making Test; AVLT, Auditory Verbal Learning Test*.

## Discussion

Several key findings were obtained from this case-control study which assessed the relationships among global *O*-GlcNAcylation, tau phosphorylation levels and MCI in T2DM subjects. (1) Global *O*-GlcNAcylation level was significantly decreased, whereas tau phosphorylation levels were increased in T2DM with MCI subjects compared with those with normal cognition. (2) High HbA1c was an independent risk factor for MCI, whereas increased *O*-GlcNAc/p-T212 was an independent protective factor for MCI in patients with T2DM; (3) *O*-GlcNAc/p-T212 was positively associated with overall cognitive function, especially with delayed learning and memory functions.

In the current study, we first performed a correlation study between global *O*-GlcNAcylation level, tau phosphorylation levels and cognitive functions in the whole blood of patients of T2DM and observed a decreased global *O*-GlcNAcylation level but increased tau phosphorylation levels in T2DM with MCI subjects. These findings were consistent with those obtained in human postmortem brain tissues, which demonstrated that protein *O*-GlcNAcylation level in AD brain was lower than that in controls (Liu F. et al., 2004). Previous study also revealed an imbalance between tau *O*-GlcNAcylation and phosphorylation in the hippocampus of a mouse model of AD (Gatta et al., 2016). In addition, *O*-GlcNAcylation elevation via the OGA inhibitor can lead to a significant reduction of pathological tau and then provides protection against neuron loss in animal studies (Yuzwa et al., 2012; Graham et al., 2014; Hastings et al., 2017). Furthermore, in accordance with our study, plasma tau levels were found higher in MCI subjects compared with cognitively normal controls in the population-based Mayo Clinic Study of Aging (Dage et al., 2016). However, our data were contradictory with the results of several studies performed on patients with AD and MCI. The plasma levels of total tau decreased among subjects with MCI and AD compared with cognitively normal controls (Sparks et al., 2012). In another study exploring the utility of plasma tau as diagnostic markers for MCI and AD, Zetterberg et al. reported that plasma tau levels were significantly elevated in AD but not in MCI compared with CN subjects (Zetterberg et al., 2013). The inconsistency in these findings may be attributed to differences in disease populations (T2DM with MCI or MCI), sample source (whole blood or plasma) and detection method of tau protein (Western blot or enzyme-linked immunosorbent assay).

High HbA1c was an independent risk factor for MCI in our T2DM subjects, which was consistent with the result reported in the action to control cardiovascular risk in diabetes-memory in diabetes (ACCORD-MIND) trial. The study indicated that higher HbA1c levels were associated with lower cognitive function in individuals with diabetes (Tali et al., 2009). When adjusted for age, sex and education, a 1% higher HbA1c level was associated with a 0.09-point lower MoCA score in non-demented elderly patients with type 2 diabetes (Huang et al., 2015). In patients with newly diagnosed T2DM, higher HbA1c was also associated with worse cognitive performances assessed by the modified 13-item version of the telephone interview for cognitive status (TICS-M) (Moulton et al., 2016). On the contrary, increased *O*-GlcNAc/p-T212 was an independent protective factor for MCI in patients with T2DM. Furthermore, *O*-GlcNAc/p-T212 was positively associated with overall cognitive performances, especially with learning and memory functions. The exact mechanisms for these relationships are not fully understood. One possible reason is that *O*-GlcNAcylation level was shown to correlate negatively with tau phosphorylation levels, which are a primary regulator of neuronal functions, including regulating long-term synaptic plasticity and learning and memory (Anggono and Huganir, 2012; Luscher and Malenka, 2012). With regard to specific phosphorylation sites of tau protein, Alonso et al. (2010) suggested that a single phosphorylation site alone had little influence on the biological activity of tau protein, while phosphorylation at Thr212 along with a modification on the C-terminal of the protein could facilitate tau aggregation. In addition, *O*-GlcNAc modification is highly abundant in the mammalian brain, especially with markedly dense expression in the hippocampus (Liu K. et al., 2004), whereas the hippocampus is mainly responsible for learning and memory function in rodents (Lazarov and Hollands, 2016). Moreover, *O*-GlcNAcylation has been linked to regulate protein homeostasis, which is essential to maintain synaptic contacts and memory (Akan et al., 2018). Xie et al. (2016) also reported that *O*-GlcNAcylation downregulation suppresses protein kinase A (PKA)-cAMP-response element binding protein signaling and consequently causes learning and memory deficits in AD. By contrast, increasing the levels of *O*-GlcNAcylation by caloric restriction can lessen learning impairment associated with diabetes (Jeon et al., 2016). Therefore, all the above explanations supported our finding that increased *O*-GlcNAc/p-T212 was a protective factor and associated with lower MCI incidence in T2DM subjects.

Our study was the first to investigate the association among *O*-GlcNAcylation level, tau phosphorylation levels, and cognitive performances in T2DM patients with MCI. Moreover, the samples used in our study were peripheral blood cells other than cerebrospinal fluid (CSF) or brain tissues, which were easier to obtain and generalize to the clinic. However, certain limitations should be noted in this study. First, due to the lack of specific *O*-GlcNAcylation antibody for tau protein, we couldn’t obtain tau *O*-GlcNAcylation level. We are also unable to obtain the level of UDP-GlcNAc because of the difficulties of detection methods (High-performance liquid chromatography/Quadrupole-Time of flight-Mass spectrometry) (Oikari et al., 2018), which could directly reflect the associations among blood glucose, key enzymes and tau *O*-GlcNAcylation level. Moreover, the relatively small sample size and sample composition of this study limited the interpretation of our results to a certain degree. In addition, this is a case-control study, and findings derived from this work cannot elucidate the direction of the relationship and it is not possible to determine the causality.

## Conclusion

Our study suggests that increased ratio of global *O*-GlcNAcylation to tau phosphorylation at Thr212 site in the whole blood is associated with decreased risk of MCI, especially with better learning and memory function in T2DM subjects. Further prospective studies with a substantial sample size should be conducted to validate these observations.

## Author Contributions

SW and RH contributed to study conception and design. RH, ST, RC, HL, DG, and JW acquired the data. RH and ST performed the analyses. RH wrote the first draft. SW, JH, and ST revised it critically for important intellectual content. All authors approved the final version to be published.

## Conflict of Interest Statement

The authors declare that the research was conducted in the absence of any commercial or financial relationships that could be construed as a potential conflict of interest.

## References

[B1] Akan I. Olivier-Van Stichelen S. Bond M. R. Hanover J. A. (2018). Nutrient-driven O-GlcNAc in proteostasis and neurodegeneration. *J. Neurochem.* 144 7–34. 10.1111/jnc.14242 29049853 PMC5735008

[B2] Alagiakrishnan K. Zhao N. Mereu L. Senior P. Senthilselvan A. (2013). Montreal cognitive assessment is superior to standardized mini-mental status exam in detecting mild cognitive impairment in the middle-aged and elderly patients with type 2 diabetes mellitus. *Biomed. Res. Int.* 2013:186106. 10.1155/2013/186106 23936778 PMC3726014

[B3] Albert M. S. DeKosky S. T. Dickson D. Dubois B. Feldman H. H. Fox N. C. (2011). The diagnosis of mild cognitive impairment due to alzheimer’s disease: recommendations from the national institute on aging-alzheimer’s association workgroups on diagnostic guidelines for alzheimer’s disease. *Alzheimers Dement.* 7 270–279. 10.1016/j.jalz.2011.03.008 21514249 PMC3312027

[B4] Alberti K. G. Zimmet P. Z. (1998). Definition, diagnosis and classification of diabetes mellitus and its complications. Part 1: diagnosis and classification of dia-betes mellitus provisional report of a WHO consultation. *Diabet. Med.* 15 539–553. 10.1002/(SICI)1096-9136(199807)15:7<539::AID-DIA668>3.0.CO;2-S 9686693

[B5] Alonso A. D. Di Clerico J. Li B. Corbo C. P. Alaniz M. E. Grundke-Iqbal I. (2010). Phosphorylation of tau at Thr212, Thr231, and Ser262 combined causes neurodegeneration. *J. Biol. Chem.* 285 30851–30860. 10.1074/jbc.M110.110957 20663882 PMC2945578

[B6] Anggono V. Huganir R. L. (2012). Regulation of AMPA receptor trafficking and synaptic plasticity. *Curr. Opin. Neurobiol.* 22 461–469. 10.1016/j.conb.2011.12.006 22217700 PMC3392447

[B7] Baglietto-Vargas D. Shi J. Yaeger D. M. Ager R. LaFerla F. M. (2016). Diabetes and alzheimer’s disease crosstalk. *Neurosci. Biobehav. Rev.* 64 272–287. 10.1016/j.neubiorev.2016.03.005 26969101

[B8] Cheng G. Huang C. Deng H. Wang H. (2012). Diabetes as a risk factor for dementia and mild cognitive impairment: a meta-analysis of longitudinal studies. *Intern. Med. J.* 42 484–491. 10.1111/j.1445-5994.2012.02758.x 22372522

[B9] Ciudin A. Espinosa A. Simo-Servat O. Ruiz A. Alegret M. Hernandez C. (2017). Type 2 diabetes is an independent risk factor for dementia conversion in patients with mild cognitive impairment. *J. Diabetes Compl.* 31 1272–1274. 10.1016/j.jdiacomp.2017.04.018 28545893

[B10] Dage J. L. Wennberg A. M. V. Airey D. C. Hagen C. E. Knopman D. S. Machulda M. M. (2016). Levels of tau protein in plasma are associated with neurodegeneration and cognitive function in a population-based elderly cohort. *Alzheimers Dement.* 12 1226–1234. 10.1016/j.jalz.2016.06.001 27436677 PMC5148697

[B11] Gao Y. Xiao Y. Miao R. Zhao J. Cui M. Huang G. (2016). The prevalence of mild cognitive impairment with type 2 diabetes mellitus among elderly people in china: a cross-sectional study. *Arch. Gerontol. Geriatr.* 62 138–142. 10.1016/j.archger.2015.09.003 26381432

[B12] Gatta E. Lefebvre T. Gaetani S. dos Santos M. Marrocco J. Mir A. M. (2016). Evidence for an imbalance between tau O-GlcNAcylation and phosphorylation in the hippocampus of a mouse model of alzheimer’s disease. *Pharmacol. Res.* 105 186–197. 10.1016/j.phrs.2016.01.006 26816085

[B13] Graham D. L. Gray A. J. Joyce J. A. Yu D. O’Moore J. Carlson G. A. (2014). Increased O-GlcNAcylation reduces pathological tau without affecting its normal phosphorylation in a mouse model of tauopathy. *Neuropharmacology* 79 307–313. 10.1016/j.neuropharm.2013.11.025 24326295

[B14] Grundke-Iqbal I. Iqbal K. Tung Y. C. Quinlan M. Wisniewski H. M. Binder L. I. (1986). Abnormal phosphorylation of the microtubule-associated protein tau (tau) in alzheimer cytoskeletal pathology. *Proc. Natl. Acad. Sci. U. S. A.* 83 4913–4917. 10.1073/pnas.83.13.4913 3088567 PMC323854

[B15] Hart G. W. Slawson C. Ramirez-Correa G. Lagerlof O. (2011). Cross talk between O-GlcNAcylation and phosphorylation: roles in signaling, transcription, and chronic disease. *Annu. Rev. Biochem.* 80 825–858. 10.1146/annurev-biochem-060608-102511 21391816 PMC3294376

[B16] Hastings N. B. Wang X. Song L. Butts B. D. Grotz D. Hargreaves R. (2017). Inhibition of O-GlcNAcase leads to elevation of O-GlcNAc tau and reduction of tauopathy and cerebrospinal fluid tau in rTg4510 mice. *Mol. Neurodegener.* 12:39. 10.1186/s13024-017-0181-0 28521765 PMC5437664

[B17] Huang L. Yang L. Shen X. Yan S. (2015). Relationship between glycated hemoglobin a1c and cognitive function in nondemented elderly patients with type 2 diabetes. *Metab. Brain Dis.* 31 347–353. 10.1007/s11011-015-9756-z 26530222

[B18] Jeon B. T. Heo R. W. Jeong E. A. Yi C. O. Lee J. Y. Kim K. E. (2016). Effects of caloric restriction on O-GlcNAcylation, Ca(2+) signaling, and learning impairment in the hippocampus of ob/ob mice. *Neurobiol. Aging* 44 127–137. 10.1016/j.neurobiolaging.2016.05.002 27318140

[B19] Kim C. Nam D. W. Park S. Y. Song H. Hong H. S. Boo J. H. (2013). O-linked beta-N-acetylglucosaminidase inhibitor attenuates beta-amyloid plaque and rescues memory impairment. *Neurobiol. Aging* 34 275–285. 10.1016/j.neurobiolaging.2012.03.001 22503002

[B20] Kim D. J. Yu J. H. Shin M. S. Shin Y. W. Kim M. S. (2016). Hyperglycemia reduces efficiency of brain networks in subjects with type 2 diabetes. *PLoS One* 11:e0157268. 10.1371/journal.pone.0157268 27336309 PMC4918925

[B21] Kim J. D. Kang S. J. Lee M. K. Park S. E. Rhee E. J. Park C. Y. (2016). C-peptide-based index is more related to incident type 2 diabetes in non-diabetic subjects than insulin-based index. *Endocrinol. Metab.* 31 320–327. 10.3803/EnM.2016.31.2.320 27349701 PMC4923417

[B22] Lazarov O. Hollands C. (2016). Hippocampal neurogenesis: learning to remember. *Prog. Neurobiol.* 13 1–18. 10.1016/j.pneurobio.2015.12.006 26855369 PMC4828289

[B23] Li W. Wang T. Xiao S. (2016). Type 2 diabetes mellitus might be a risk factor for mild cognitive impairment progressing to alzheimer’s disease. *Neuropsychiatr. Dis. Treat.* 12 2489–2495. 10.2147/NDT.S111298 27729793 PMC5047733

[B24] Liu F. Iqbal K. Grundke-Iqbal I. Hart G. W. Gong C. X. (2004). O-GlcNAcylation regulates phosphorylation of tau: a mechanism involved in alzheimer’s disease. *Proc. Natl. Acad. Sci. U. S. A.* 101 10804–10809. 10.1073/pnas.0400348101 15249677 PMC490015

[B25] Liu K. Paterson A. J. Zhang F. McAndrew J. Fukuchi K. Wyss J. M. (2004). Accumulation of protein O-GlcNAc modification inhibits proteasomes in the brain and coincides with neuronal apoptosis in brain areas with high O-GlcNAc metabolism. *J. Neurochem.* 89 1044–1055. 10.1111/j.1471-4159.2004.02389.x 15140202

[B26] Luscher C. Malenka R. C. (2012). NMDA receptor-dependent long-term potentiation and long-term depression (LTP/LTD). *Cold Spring Harb. Perspect. Biol.* 4:a005710. 10.1101/cshperspect.a005710 22510460 PMC3367554

[B27] Ma L. Wang J. Li Y. (2015). Insulin resistance and cognitive dysfunction. *Clin. Chim. Acta.* 444 18–23. 10.1016/j.cca.2015.01.027 25661087

[B28] Mehta H. B. Mehta V. Goodwin J. S. (2017). Association of hypoglycemia with subsequent dementia in older patients with type 2 diabetes mellitus. *J. Gerontol. A. Biol. Sci. Med. Sci.* 72 1110–1116. 10.1093/gerona/glw217 27784724 PMC5861972

[B29] Mittal K. Katare D. P. (2016). Shared links between type 2 diabetes mellitus and alzheimer’s disease: a review. *Diabetes Metab. Syndr.* 10(2 Suppl. 1) S144–S149. 10.1016/j.dsx.2016.01.021 26907971

[B30] Moulton C. D. Stewart R. Amiel S. A. Laake J. P. Ismail K. (2016). Factors associated with cognitive impairment in patients with newly diagnosed type 2 diabetes: a cross-sectional study. *Aging Ment. Health* 20 840–847. 10.1080/13607863.2015.1040723 25959123

[B31] Myslicki J. P. Shearer J. Hittel D. S. Hughey C. C. Belke D. D. (2014). O-GlcNAc modification is associated with insulin sensitivity in the whole blood of healthy young adult males. *Diabetol. Metab. Syndr.* 6:96. 10.1186/1758-5996-6-96 25228926 PMC4164748

[B32] Nasreddine Z. S. Phillips N. A. Bedirian V. Charbonneau S. Whitehead V. Collin I. (2005). The montreal cognitive assessment, MoCA: a brief screening tool for mild cognitive impairment. *J. Am. Geriatr. Soc.* 53 695–699. 10.1111/j.1532-5415.2005.53221.x 15817019

[B33] Ogurtsova K. da Rocha Fernandes J. D. Huang Y. Linnenkamp U. Guariguata L. Cho N. H. (2017). IDF diabetes atlas: global estimates for the prevalence of diabetes for 2015 and 2040. *Diabetes Res. Clin. Pract.* 128 40–50. 10.1016/j.diabres.2017.03.024 28437734

[B34] Oikari S. Kettunen T. Tiainen S. Hayrinen J. Masarwah A. Sudah M. (2018). UDP-sugar accumulation drives hyaluronan synthesis in breast cancer. *Matrix Biol.* 67 63–74. 10.1016/j.matbio.2017.12.015 29331336

[B35] Park K. Saudek C. D. Hart G. W. (2010). Increased expression of beta-N-acetylglucosaminidase in erythrocytes from individuals with pre-diabetes and diabetes. *Diabetes* 59 1845–1850. 10.2337/db09-1086 20413512 PMC2889787

[B36] Peterson S. B. Hart G. W. (2016). New insights: a role for O-GlcNAcylation in diabetic complications. *Crit. Rev. Biochem. Mol. Biol.* 51 150–161. 10.3109/10409238.2015.1135102 26806492

[B37] Sheen Y. J. Sheu W. H. (2016). Association between hypoglycemia and dementia in patients with type 2 diabetes. *Diabetes Res. Clin. Pract.* 116 279–287. 10.1016/j.diabres.2016.04.004 27321346

[B38] Sparks D. L. Kryscio R. J. Sabbagh M. N. Ziolkowski C. Lin Y. Sparks L. M. (2012). Tau is reduced in AD plasma and validation of employed ELISA methods. *Am. J. Neurodegener. Dis.* 1 99–106. 23383382 PMC3560452

[B39] Springhorn C. Matsha T. E. Erasmus R. T. Essop M. F. (2012). Exploring leukocyte O-GlcNAcylation as a novel diagnostic tool for the earlier detection of type 2 diabetes mellitus. *J. Clin. Endocrinol. Metab.* 97 4640–4649. 10.1210/jc.2012-2229 23066116

[B40] Tali C. Y. Hertzel C. G. Jefe D. W. Ronald M. L. Michael E. M. Laura H. C. (2009). Relationship between baseline glycemic control and cognitive function in individuals with type 2 diabetes and other cardiovascular risk factors. *Diabetes Care* 32 221–226. 10.2337/dc08-1153 19171735 PMC2628683

[B41] Tiermey M. C. Szalai J. C. Snow W. G. Fisher R. H. Nores A. Nadon G. (1996). Prediction of probable alzheimer’s disease in memory-impaired patients: a prospective longitudinal study. *Neurology* 46 661–665. 10.1212/WNL.46.3.661 8618663

[B42] Wang Z. Park K. Comer F. Hsieh-Wilson L. C. Saudek C. D. Hart G. W. (2009). Site-specific GlcNAcylation of human erythrocyte proteins: potential biomarker(s) for diabetes. *Diabetes* 58 309–317. 10.2337/db08-0994 18984734 PMC2628603

[B43] Xie S. Jin N. Gu J. Shi J. Sun J. Chu D. (2016). O-GlcNAcylation of protein kinase a catalytic subunits enhances its activity: a mechanism linked to learning and memory deficits in alzheimer’s disease. *Aging Cell* 15 455–464. 10.1111/acel.12449 26840030 PMC4854926

[B44] Yang X. Qian K. (2017). Protein O-GlcNAcylation: emerging mechanisms and functions. *Nat. Rev. Mol. Cell Biol.* 18 452–465. 10.1038/nrm.2017.22 28488703 PMC5667541

[B45] Yuzwa S. A. Shan X. Jones B. A. Zhao G. Woodward M. L. Li X. (2014). Pharmacological inhibition of O-GlcNAcase (OGA) prevents cognitive decline and amyloid plaque formation in bigenic tau/APP mutant mice. *Mol. Neurodegener.* 9:42. 10.1186/1750-1326-9-42 25344697 PMC4232697

[B46] Yuzwa S. A. Shan X. Macauley M. S. Clark T. Skorobogatko Y. Vosseller K. (2012). Increasing O-GlcNAc slows neurodegeneration and stabilizes tau against aggregation. *Nat. Chem. Biol.* 8 393–399. 10.1038/nchembio.797 22366723

[B47] Zachara N. Akimoto Y. Hart G. W. (2015). “The O-GlcNAc Modification,” in *Essentials of Glycobiology* eds Varki A. Cummings R. D. Esko J. D. Stanley P. Hart G. W. Aebi M. (New York, NY: Cold Spring Harbor) 239–251.

[B48] Zetterberg H. Wilson D. Andreasson U. Minthon L. Blennow K. Randall J. (2013). Plasma tau levels in alzheimer’s disease. *Alzheimers Res. Ther.* 5:9. 10.1186/alzrt163 23551972 PMC3707015

[B49] Zheng Y. Ley S. H. Hu F. B. (2018). Global aetiology and epidemiology of type 2 diabetes mellitus and its complications. *Nat. Rev. Endocrinol.* 14 88–98. 10.1038/nrendo.2017.151 29219149

